# Supplementary material for: Operando recombination kinetics in perovskite nanocrystal films revealed by in situ time-resolved photoluminescence
Source: Nat Commun. 2025 Dec 12;16:11352. doi: 10.1038/s41467-025-66414-3 (PMC12728232; doi:10.1038/s41467-025-66414-3)
Supplement: Supplementary file 1 — Supplementary Information [file 41467_2025_66414_MOESM1_ESM.docx]

Supporting Information

Operando Recombination Kinetics in Perovskite Nanocrystal Films Revealed by In-Situ Time-Resolved Photoluminescence

Dandan Cao^1^, Ziyue Jiao^1^, Jie Gao^1^, Yi Wang^1^*, Xi-Cheng Ai^1^, Jian-Ping Zhang^1^

**Affiliations**

^1^Key Laboratory of Advanced Light Conversion Materials and Biophotonics, School of Chemistry and Life Resources
Renmin University of China, Beijing 100872, P.R. China

**S1. Particle size distribution obtained from the transmission electron microscopy image**

**
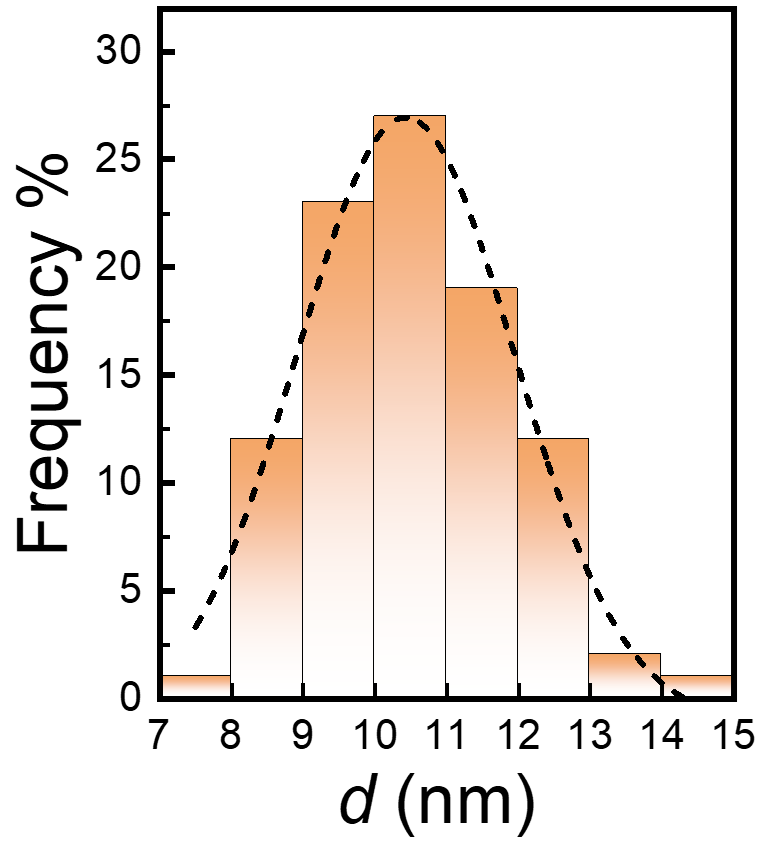
**

**Supplementary Fig. 1.** Histogram of the size distribution for PNCs obtained from the TEM image, which is fitted with a Gaussian function yielding an average particle size of ~10.4 nm.

**S2. Particle size distribution obtained from the dynamic light scattering (DLS) results**


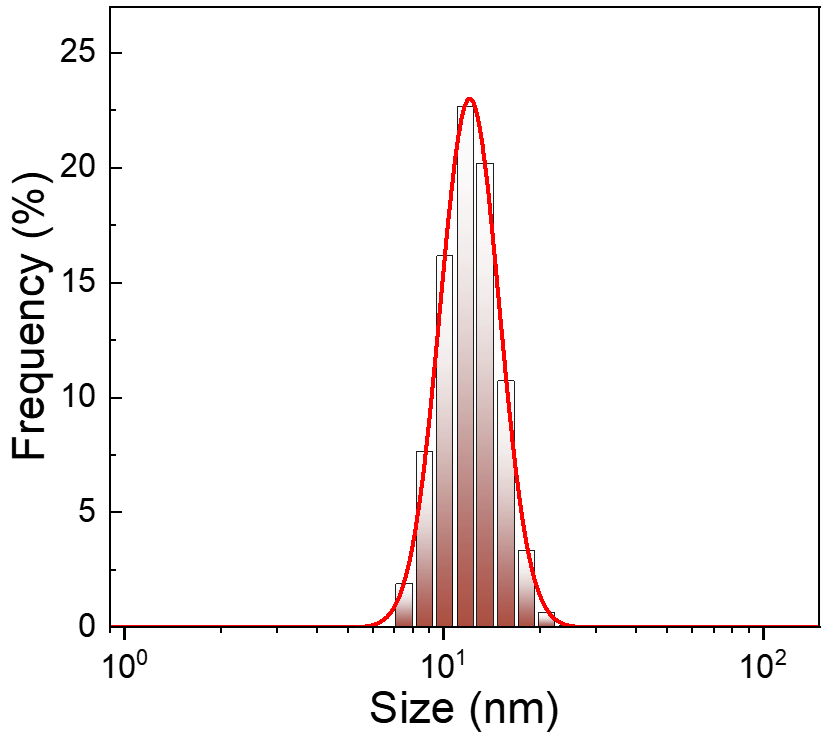


**Supplementary Fig. 2.** Histogram of the size distribution for PNCs obtained from the DLS results, which is fitted with a Gaussian function yielding an average particle size of ~12 nm

**S3. Thickness determination of PNC films**


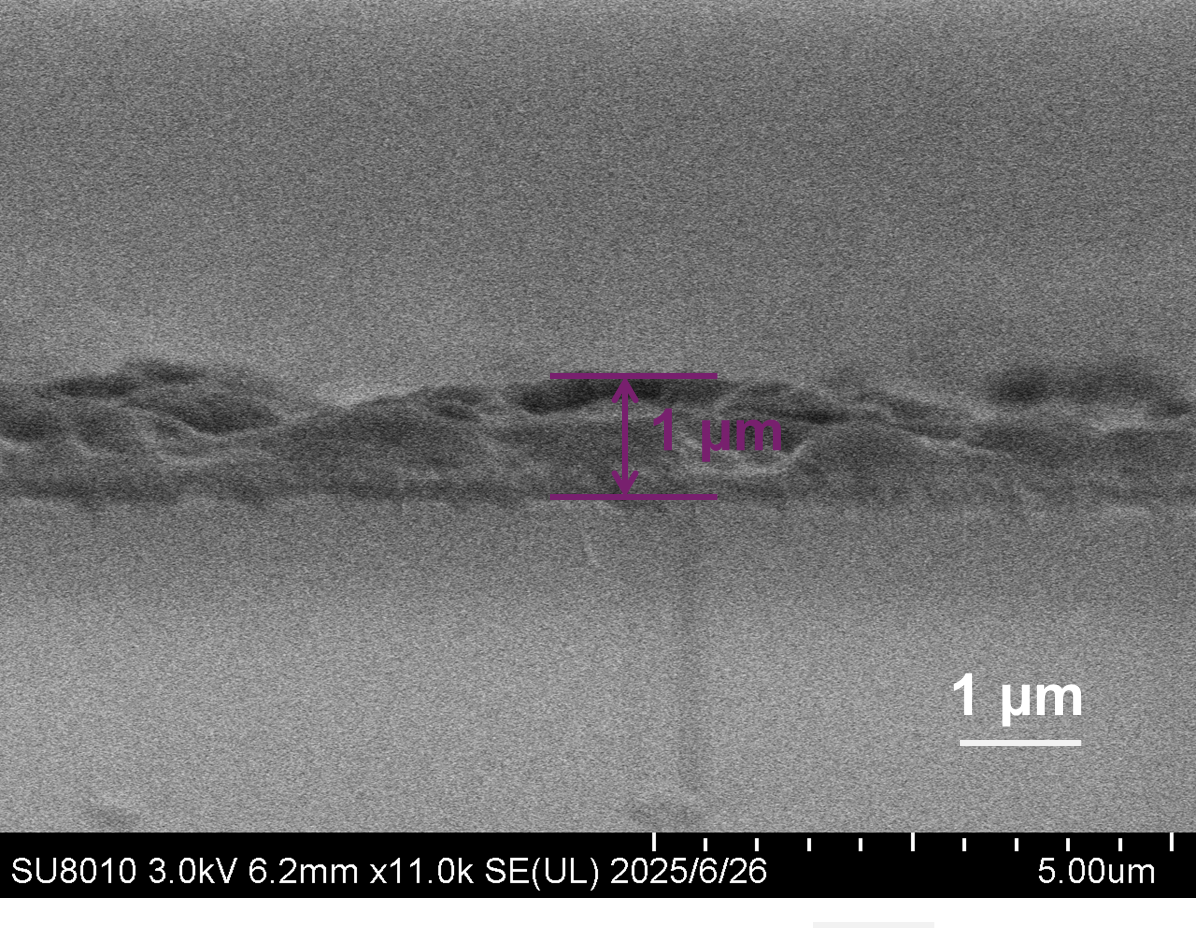

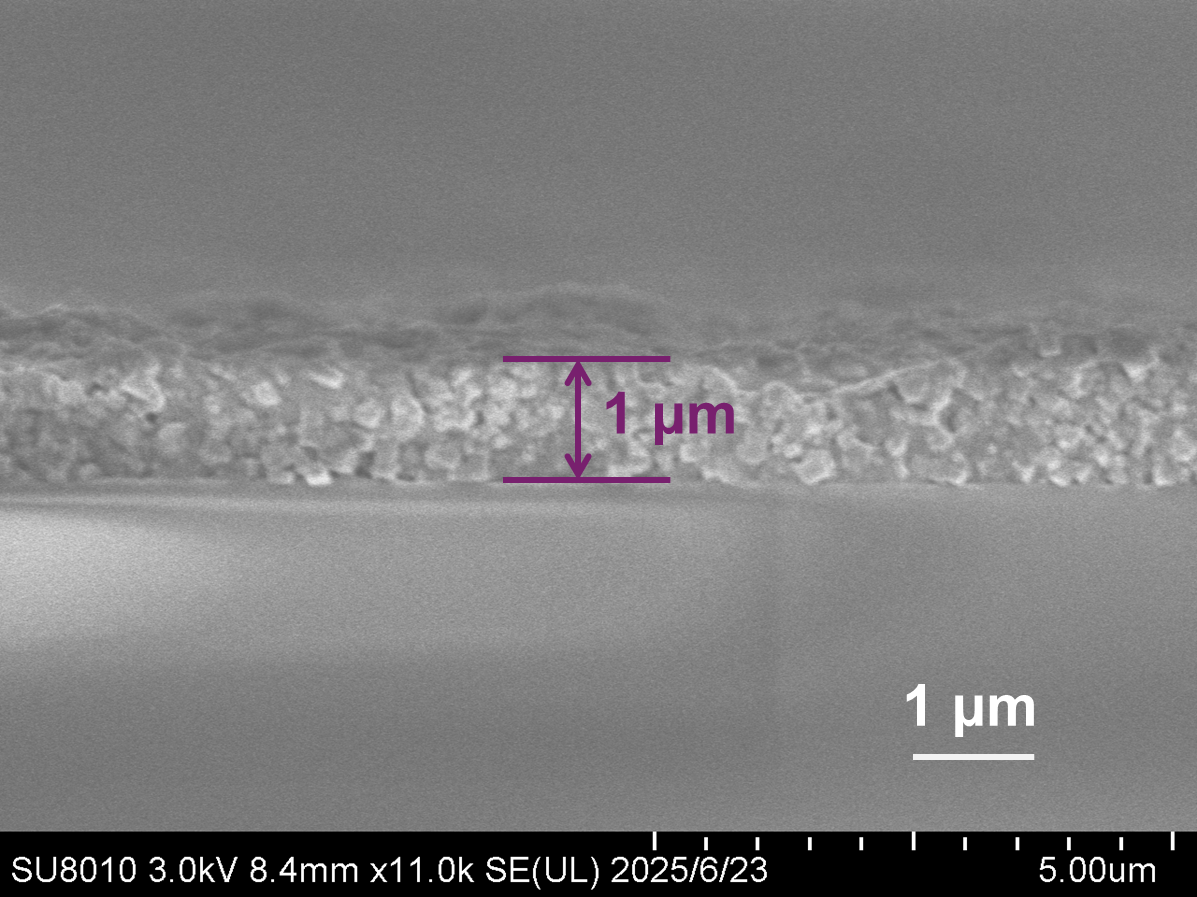


**Supplementary Fig. 3.** Cross-sectional scanning electron microscopy images of unannealed (left) and annealed (right) PNC films, both of which exhibit a uniform thickness of ~1 μm as denoted by the arrows.

**S4. Characterization of PL homogeneity for PNC films**

**Supplementary Fig. 4.** PL microscopy images of CsPbBr_3_ PNC films without and with the annealing treatment demonstrating homogeneous PL emission within the entire excitation regions.

**S5.** **PL quantum yield (PLQY) determination of PNC films**


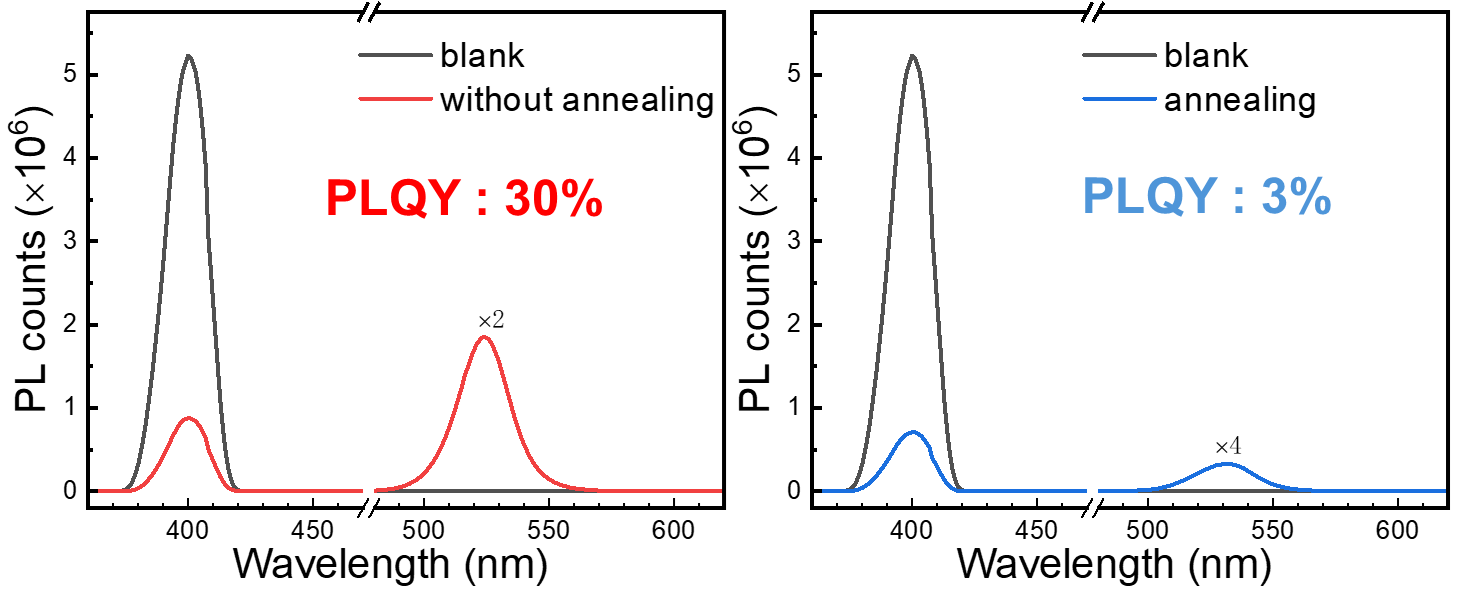


**Supplementary Fig. 5.** Representative data of PLQY determination by a fluorescence spectrometer equipped with an integrating sphere. The black lines correspond to the spectra of the excitation beams without samples, while the red and blue lines display spectra of both scattered excitation light and PL emission for unannealed (left) and annealed (right) PNC films. The PLQY value is determined by calculating the ratio of the integrated area of the PL spectra to that of the reduced excitation light spectra.

**S6.** **PL spectra of the PNC films before and after the preillumination treatment**


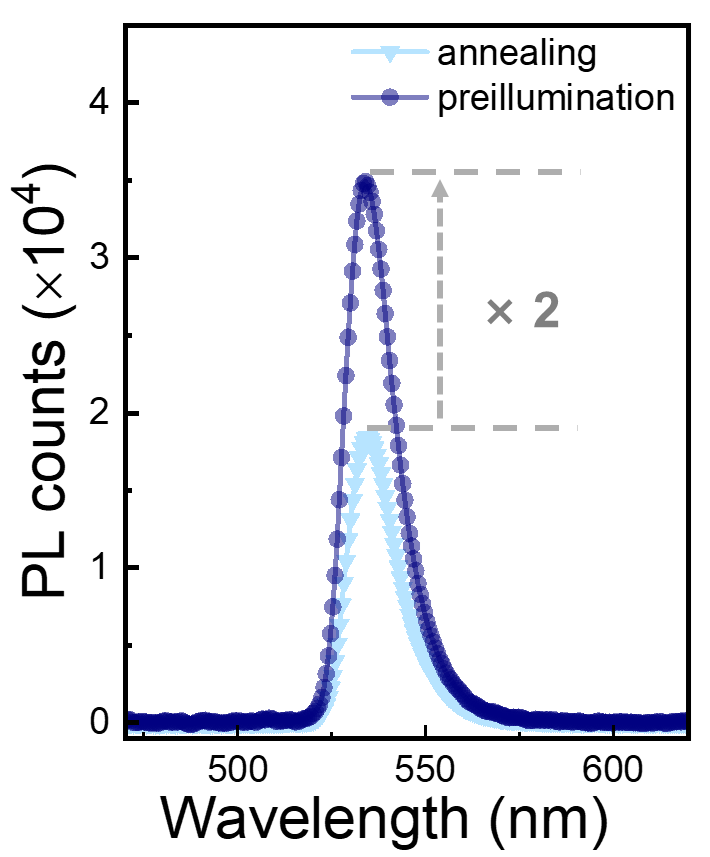


**Supplementary Fig. 6.** Comparison between the PL spectra of the annealed PNC films before and after a preillumination treatment, which exhibits an approximately twofold enhancement in the PL intensity.

**S7. In-situ PL spectra of the PNC films with different treatments**


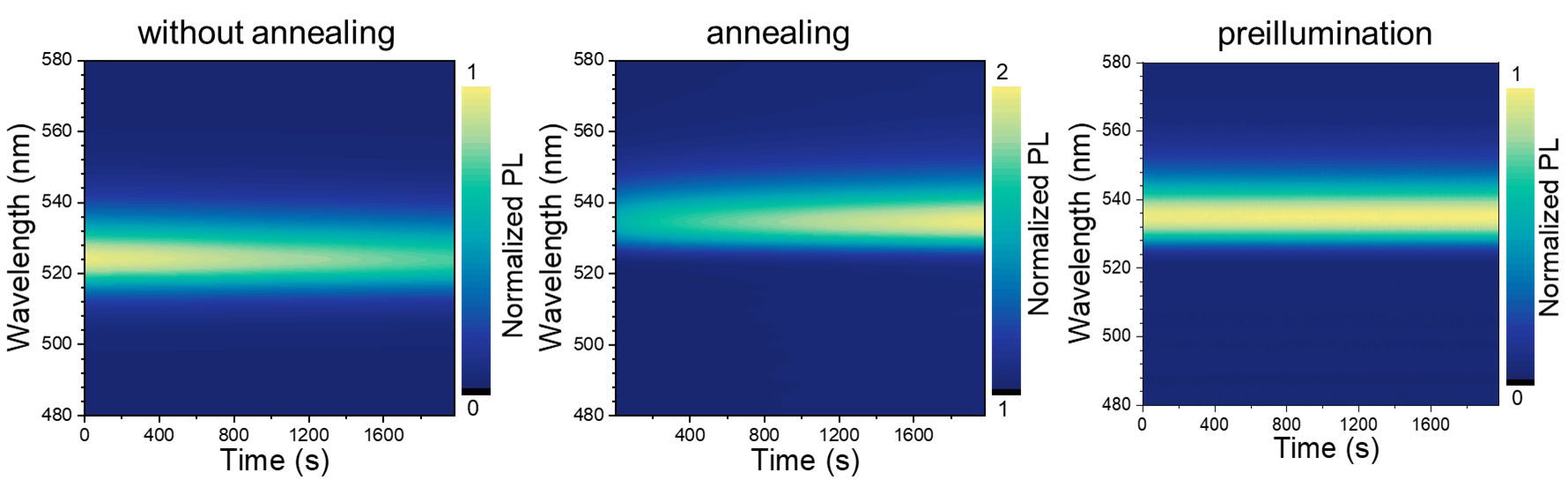


**Supplementary Fig. 7.** Evolution of the PL spectra for PNC films with different treatments (corresponding to Figure 2f of the main text) under continuous CW illumination, where the maximum of the PL intensity has been normalized.

**S8. Stability examination of the integrated in-situ PL spectroscopy platform**


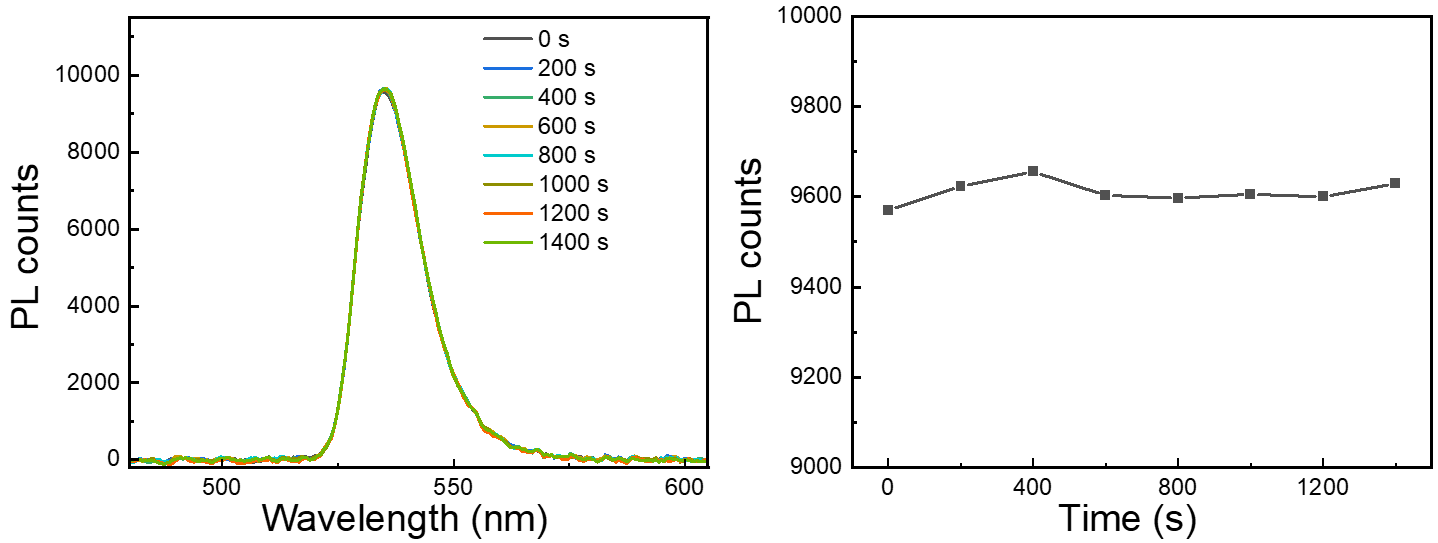


**Supplementary Fig. 8.** (a) Steady-state PL spectra and (b) the corresponding peak counts of the PNC film measured by the integrated in-situ PL spectroscopy platform with different illumination durations (corresponding to Figure 3b in the main text). The spectral profiles are well preserved, and the fluctuation of the peak counts is suppressed to be less than 2%.

**S9** **In-situ TRPL spectra of the PNC films during one testing period**

**
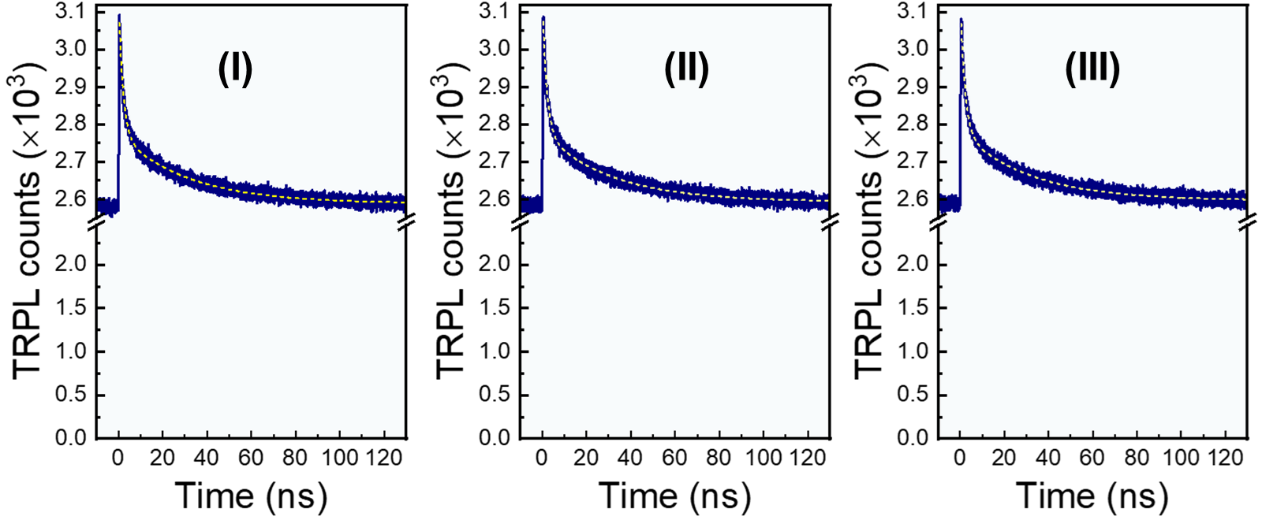
**

**Supplementary Fig. 9.** In-situ TRPL spectra of the CsPbBr₃ PNC film recorded during three successive periods (I-III) at a constant CW pump intensity of 6.37 mW/cm^2^ (see experimental details in Figure 3 of the main text).

**Supplementary Table 1.** Fitting parameters extracted from the in-situ TRPL spectra shown in Supplementary Figure 9. All the TRPL kinetics were measured under a fixed CW excitation intensity of 6.37 mW/cm² and fitted to a biexponential decay function.

| **Time** | **(I)** | **(II)** | **(III)** |
| --- | --- | --- | --- |
| $\boldsymbol{A}_{\mathbf{1}}$ | 0.62 | 0.61 | 0.61 |
| $\boldsymbol{\tau}_{\mathbf{1}} \boldsymbol{(}\mathbf{ns}\boldsymbol{)}$ | 1.99 | 2.05 | 2.15 |
| $\boldsymbol{A}_{\boldsymbol{2}}$ | 0.38 | 0.39 | 0.39 |
| $\boldsymbol{\tau}_{\mathbf{2}} \boldsymbol{(}\mathbf{ns}\boldsymbol{)}$ | 32.58 | 33.16 | 32.89 |
| $\boldsymbol{<\tau>}\boldsymbol{(}\mathbf{ns}\boldsymbol{)}$ | 13.56 | 14.06 | 14.11 |

The TRPL profiles of PNC films under different pulsed laser excitation intensities are fitted with biexponential functions following the procedure of baseline subtraction, $I\left( t \right)=A_{1}e^{-t/\tau_{1}}+A_{2}e^{-t/\tau_{2}}$, where $I(t)$ represents the time-dependent PL intensity, $A_{1}$ and $A_{2}$ are amplitude coefficients, and $\tau_{1}$ and $\tau_{2}$ correspond to the lifetimes of the two decay components. The average PL lifetimes ($<\tau>$) are thus obtained according to the expression, $<\tau>=(\sum A_{i}\tau_{i})/\sum A_{i}$.

**S10.** **Examination of the influence of the ps pulse on in-situ PL spectra measurements**


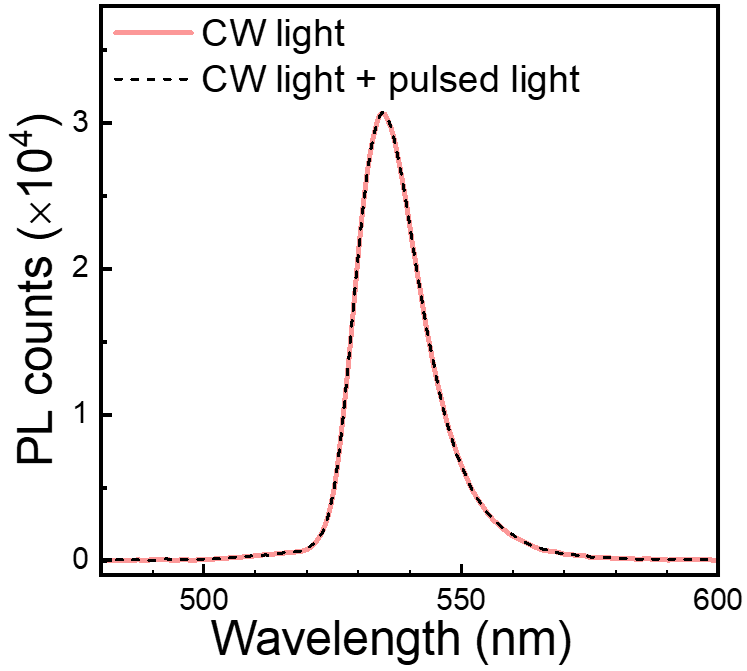


**Supplementary Fig. 10.** Steady-state PL spectra with and without the superposition of the ps pulsed laser on the CW beam. No observable changes in the PL intensity and profile confirm that the pulsed beam does not influence the operando condition, thus supporting the prerequisition of *perturbation*.

**S11. Influence of the ps pulse intensity on in-situ TRPL kinetics**


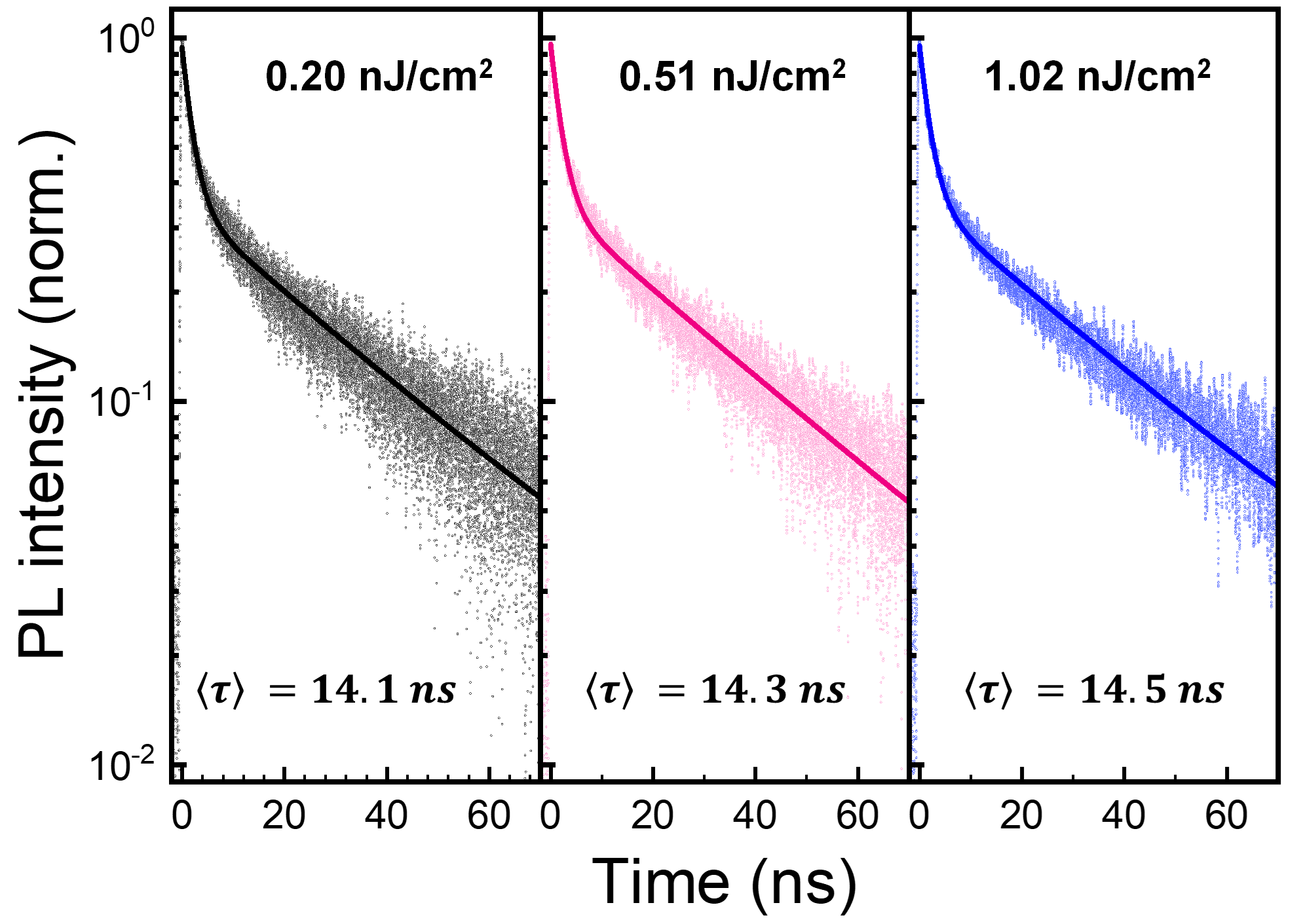


**Supplementary Fig. 11.** In-situ TRPL of PNC films as a function of pulsed excitation intensity, where the intensity of the CW beam remains unchanged (~7 mW/cm^2^). The circles are experimental data, and the solid curves are bi-exponential fitting results. The pulse intensities and as-obtained decay time constants have been displayed in the corresponding panels.

**S12. Steady-state PL spectra of PNC films under various CW pump intensities**

**Supplementary Fig. 12.** Steady-state PL spectra of the PNC films as a function of CW pump fluence. The intensities of the CW beam from bottom to top are 0.64, 1.27, 1.91, 2.55, 3.44, 4.59, 5.22, 7.13, 8.92, 9.55, 10.45, 11.97 and 12.74 mW/cm^2^, respectively.

**S13. Reproducibility examination for the in-situ steady-state PL spectra**


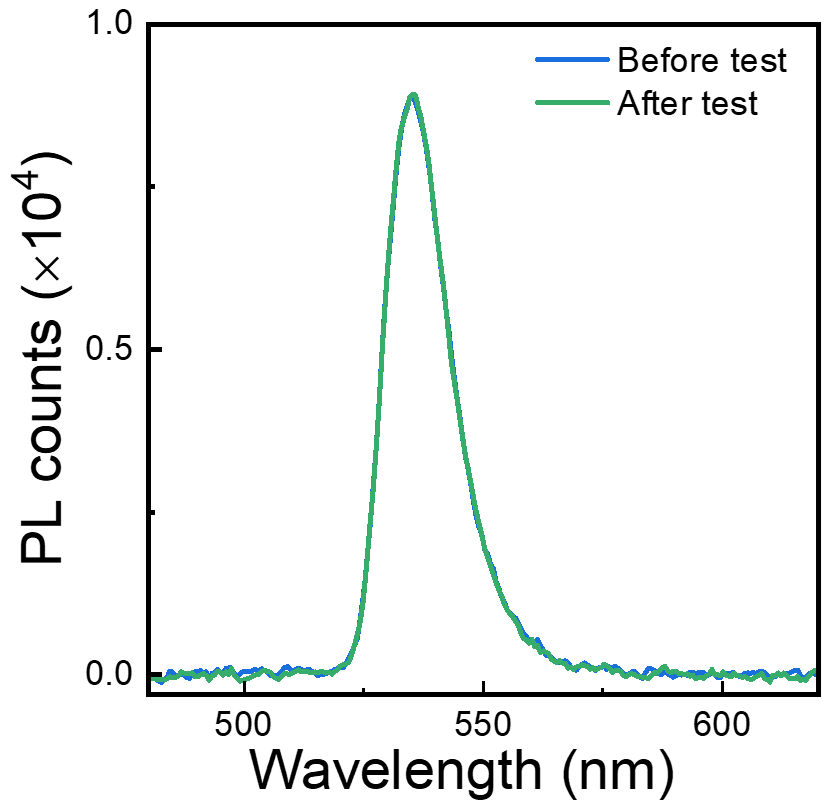


**Supplementary Fig. 13.** Steady-state PL spectra of the PNC film before and after the entire period of the in-situ spectroscopy measurements.

**S14. Reproducibility examination for the in-situ TRPL kinetics**

**Supplementary Fig. 14.** TRPL kinetics of the PNC film before and after the entire period of the in-situ spectroscopy measurements.

**S15. Comparison between two fitting models**

**Supplementary Fig. 15.** In-situ TRPL kinetics (CW intensity: 0.64 mW/cm^2^, black circles) and the fitting results based on a bi-exponential function (yellow line) and a stretched exponential function (red). The stretched exponential model displays obvious deviation at the early time delays (< 1 ns).

**S16. Fitting results of the in-situ TRPL data based on bi-exponential functions**


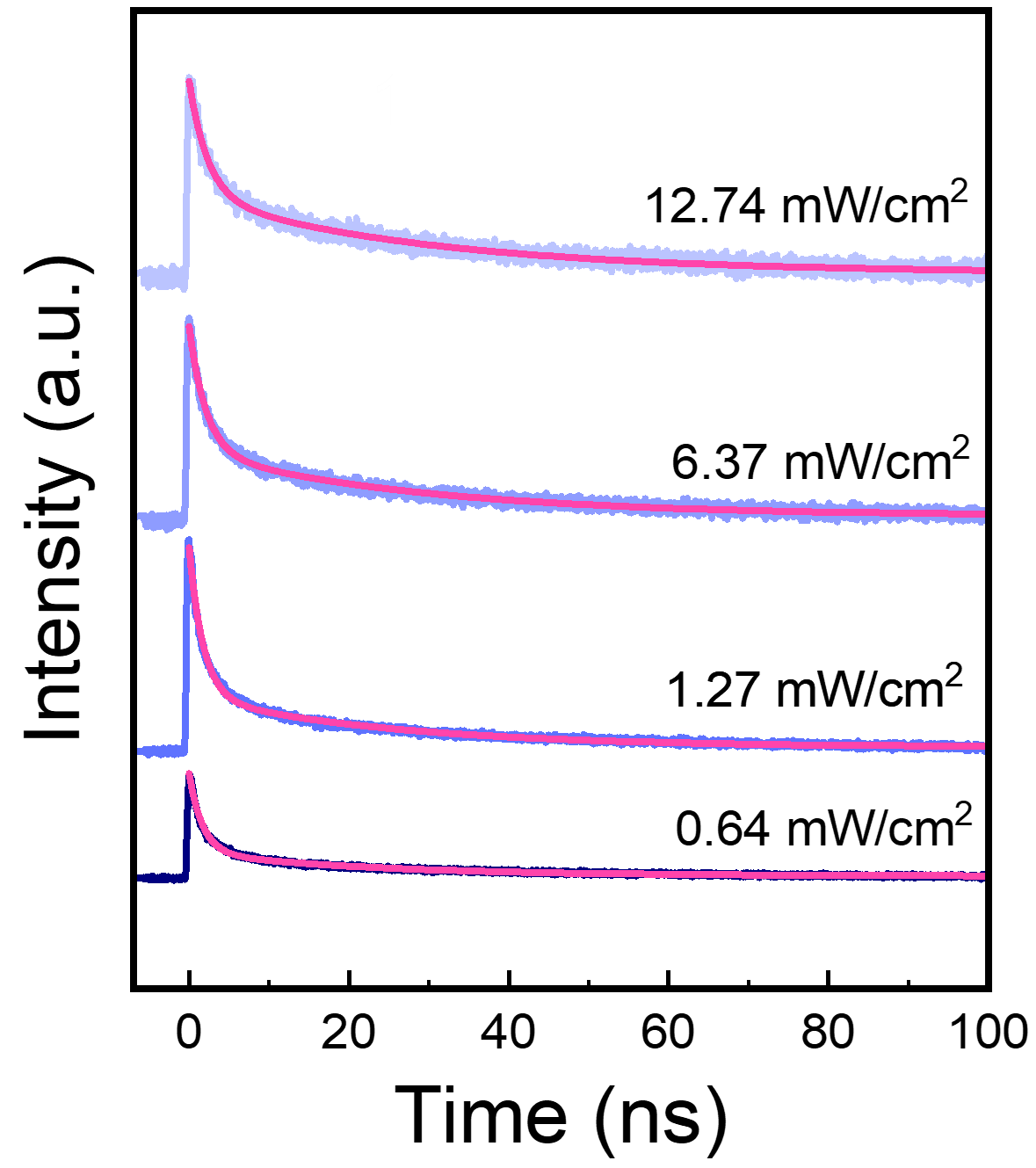


**Supplementary Fig. 16.** In-situ TRPL decay kinettics of the PNC film under different CW pump intensities fitted to the bi-exponential mode.

**Supplementary Table 2.** Fitting results of in-situ TRPL kinetics derived from the results shown in Supplementary Fig. 16.

| **Pump intensity** | **0.64 mW/cm^2^** | **1.27 mW/cm^2^** | **6.37 mW/cm^2^** | **12.74 mW/cm^2^** |
| --- | --- | --- | --- | --- |
| $\boldsymbol{A}_{\mathbf{1}}$ | 0.78 | 0.75 | 0.66 | 0.60 |
| $\boldsymbol{\tau}_{\mathbf{1}} \boldsymbol{(}\mathbf{ns}\boldsymbol{)}$ | 1.85 | 1.92 | 2.31 | 2.53 |
| $\boldsymbol{A}_{\boldsymbol{2}}$ | 0.22 | 0.25 | 0.34 | 0.40 |
| $\boldsymbol{\tau}_{\mathbf{2}} \boldsymbol{(}\mathbf{ns}\boldsymbol{)}$ | 32.70 | 33.54 | 36.87 | 35.6. |
| $\boldsymbol{<\tau>}\boldsymbol{(}\mathbf{ns}\boldsymbol{)}$ | 8.70 | 9.80 | 13.91 | 15.65 |

**S17. Micro-PL intensities as a function of excitation power and integration time**


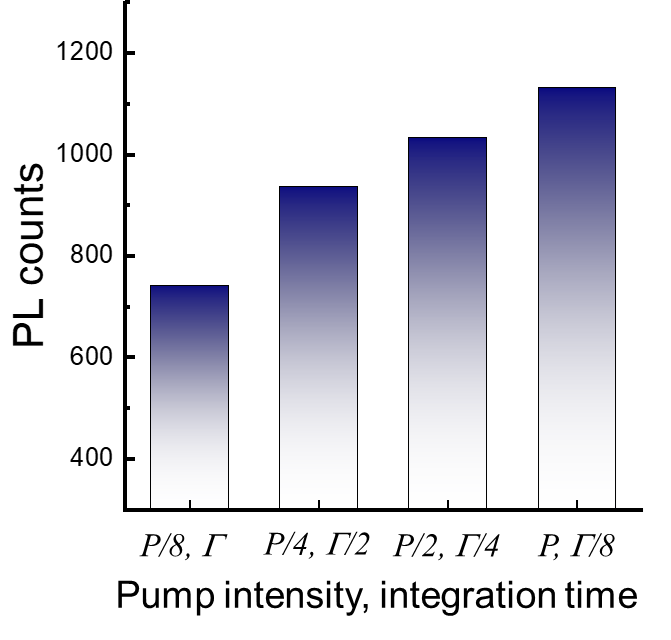


**Supplementary Fig. 17.** PL counts obtained from the wide-field PL microscopy acquired under different excitation powers and integration times, while their products remain constant.
